# ﻿New species and new records of *Phrynarachne* crab spiders (Araneae, Thomisidae) from Southeast Asia

**DOI:** 10.3897/zookeys.1257.165973

**Published:** 2025-10-24

**Authors:** Mikhail M. Omelko

**Affiliations:** 1 Federal Scientific Center of East Asia Terrestrial Biodiversity, Far Eastern Branch, Russian Academy of Sciences, Vladivostok 690022, Russia Federal Scientific Center of East Asia Terrestrial Biodiversity, Russian Academy of Sciences Vladivostok Russia

**Keywords:** Aranei, biodiversity, bird-dropping crab spiders, Borneo, Laos, Luzon, Stephanopinae, redescription, taxonomy

## Abstract

Two new species of *Phrynarachne*, *P. gorochovi***sp. nov.** (♀), from Philippines (Luzon) and *P. storozhenkoi***sp. nov.** (♀) from Malaysia (Borneo) are diagnosed, illustrated and described. Two species, *P. ceylonica* (O. Pickard-Cambridge, 1884) and *P. decipiens* Forbes, 1884 are reported from Laos for the first time and their females are redescribed in detail. The distribution of *P. decipiens* is discussed.

## ﻿Introduction

With 35 species currently known (WSC 2025), *Phrynarachne* Thorell, 1869 is a medium-sized genus of Thomisidae. Representatives of this genus are distributed across the Indomalayan region (19 species), Afrotropics (9), southeastern Palaearctic (3), and Australasia (3) (WSC 2025). These spiders are often referred to as bird-dropping crab spiders due to their remarkable mimicry as their body shape and coloration closely resemble bird droppings on leaf surfaces. To enhance this deceptive appearance, these spiders construct a thin white silk patch on a leaf and place insect remains within it; the spider itself rests in the center of this structure (Fig. 27). In addition to this striking morphology and behavior, *Phrynarachne* spiders emit a distinct foul odor that attracts insects such as flies (Forbes 1884; Yu et al. 2015).

The genus *Phrynarachne* has never been subjected to a global revision, and the available information on its species is scattered across numerous publications (Tang and Li 2010; Dippenaar-Schoeman and Wiese 2022; Dippenaar-Schoeman 2024, etc.). Overall, the genus remains poorly studied with only 11 species known from both sexes, while the others are known only from a single sex (two species from males, 20 from females) or from a juvenile specimen (1 species). Eight species are known only from text descriptions lacking illustrations. Eighteen species are poorly known, being represented only by old original descriptions and/or lacking high-quality images (WSC 2025). Among Asian representatives of the genus, the Chinese fauna is currently the best studied. Most Chinese species known to date were revised by Lin et al. (2022), who described four new species and reported the previously unknown sex for three others. Several additional studies on *Phrynarachne* species from India have been published in recent years (Das et al. 2019, 2024; Dash and Sivaperuman 2021). In contrast, the diversity of this genus in Southeast Asia remains poorly known. A number of species are still known only from their original descriptions, which often lack detailed figures. The types of only two species, *P. aspera* Thorell, 1895 and *P. bimaculata* Thorell, 1895 (both from Myanmar), have recently been reexamined and redescribed (Sherwood et al. 2022).

A study of material housed in the Bioresource Collection of the Federal Scientific Centre of East Asia Terrestrial Biodiversity, Far Eastern Branch of the Russian Academy of Sciences, has revealed four species of *Phrynarachne*, two of which are new to science. The aims of the present study are: (1) to provide detailed descriptions and diagnoses of the new species; (2) to redescribe females of *P. ceylonica* (O. Pickard-Cambridge, 1884) and *P. decipiens* Forbes, 1884; (3) to report these two species as new records for Laos; (4) to discuss the distribution of *P. decipiens*; and (5) to map the distribution of species from Indomalayan, Palaearctic, and Australasian realms based on new material and published records.

## ﻿Material and methods

Specimens were photographed using a Nikon DSRi2 camera attached to a Nikon SMZ25 stereomicroscope at the Far Eastern Federal University (Vladivostok, Russia). Photographs were taken in dishes filled with alcohol, with soft white paper at the bottom. Live specimens were photographed using Nikon D800 DSLR camera with Tamron SP 90 mm F2.8 Di VC USD Macro (F004N) lens. Digital images were montaged using ZERENE STACKER (https://zerenesystems.com/cms/stacker) software package. Epigynes were cleared in a KOH/water solution. The distribution map was produced using SIMPLEMAPPR (Shorthouse 2010). All measurements are in millimeters. Length of leg segments was measured on the retrolateral side, and is shown as: femur, patella, tibia, metatarsus, tarsus (total length). All examined material is deposited in the Zoological Museum of the Moscow State University, Moscow, Russia (ZMMU; curator K.G. Mikhailov), and in the Bioresource Collection of the Federal Scientific Centre of East Asia Terrestrial Biodiversity of the Far East Branch of the Russian Academy of Sciences (IBSS, reg. number 2797657).

Abbreviations used in the text and the format of descriptions follow Lin et al. 2022, with some modifications.

Eyes: **ALE** – anterior lateral eye, **AME** – anterior median eye, **PLE** – posterior lateral eye, **PME** – posterior median eye.

Leg segments: **Fe** – femur, **Mt** – metatarsus, **Pa** – patella, **Ti** – tibia.

Spination: **d** – dorsal, **p** – prolateral, **r** – retrolateral, **v** – ventral.

Copulatory organs: **CO** – copulatory opening, **FD** – fertilization duct, **H** – hood, **MP** – median plate, **Re** – receptacle.

## ﻿Taxonomy

### ﻿Family Thomisidae Sundevall, 1833

#### ﻿Genus Phrynarachne

Taxon classificationAnimaliaAraneaeThomisidae

Thorell, 1869

61DA99BE-FE39-5C6C-96A9-2C294F500550

##### Type species.

*Phrynarachne rugosa* (Walckenaer, 1805), from Mauritius.

##### Note.

Spiders of medium to large size, bearing large tubercles on the opisthosoma. In many species, tubercles are also found on the carapace and legs (especially large ones on legs I and II). Chelicerae with two promarginal and one retromarginal tooth. The epigyne with a simple, usually rectangular median plate, and the receptacles are strongly sclerotized. For the full genus diagnosis, see Lin et al. (2022). The genus comprises 35 described species, of which 9 occur in Africa and 26 in Asia and Australasia (WSC 2025). The genus is currently not divided into species groups. Afrotropical species (except those from South Africa) are poorly studied and are mainly known only from their original descriptions (WSC 2025). Despite their broad distribution, the species from Africa and other regions are morphologically similar and clearly belong to the same genus. Species of the genus may occur in sympatry; *P. ceylonica* and *P. decipiens* were collected by the author at the same locality on the same day.

#### ﻿ Phrynarachne gorochovi sp. nov.

Taxon classificationAnimaliaAraneaeThomisidae

5316A90D-8DC1-5E7F-ABBD-6E51BA284EB0

https://zoobank.org/942B88A2-CA73-4A3F-A615-D7509F32D8D7

Figs 1–4, 5–7, 28

##### Type material.

***Holotype*.** Philippines • ♀; Luzon Isl., Nueva Ecija Prov.; 15°39'N, 121°14'E; 370 m a.s.l.; 10–18.02.1993; A.V. Gorochov leg.; ZMMU.

##### Etymology.

The specific epithet is after Andrei Vasil’evich Gorochov (St-Petersburg, Russia), a Russian entomologist specializing in Polyneoptera taxonomy, who collected the holotype.

##### Diagnosis.

By the presence of long triangular tubercles on the lateral sides of opisthosoma, the female of the new species resembles those of *P. brevis* Tang & Li, 2010 from southern China. *Phrynarachne gorochovi* sp. nov. can be easily distinguished from this species by the median plate (MP) of the epigyne, which has an indistinct posterior margin and lateral edges directed posteriorly (vs. posterior margin distinct, lateral edges directed anteriorly; cf. Fig. 5 and Lin et al. 2022: fig. 6A).

##### Description.

**Figures 1–4.**
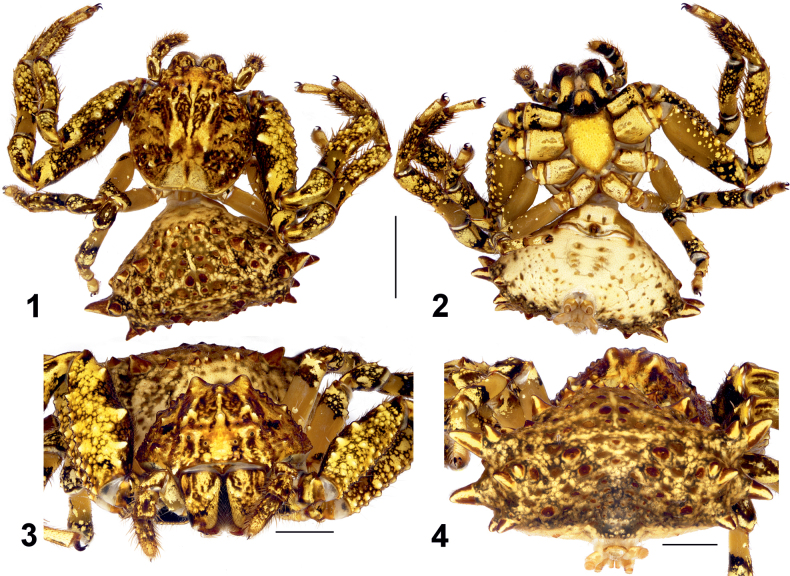
Female habitus of *Phrynarachne gorochovi* sp. nov. 1. Dorsal; 2. Ventral; 3. Anterior; 4. Posterior. Scale bars: 5 mm (1, 2); 2.5 mm (3, 4).

**Female** (Figs 1–4). Total length 15.30. Carapace 7.37 long, 7.55 wide. Opisthosoma 7.52 long, 14.36 wide. Carapace light brown with yellow median band with dark brown irregular spots; lateral sides with dark brown radial stripes; with 2 large humps at head area (Fig. 3) and numerous small tubercles across its surface. Clypeus yellow with dark brown irregular spots. Chelicerae yellow with numerous dark brown irregular spots. Labium’s proximal half yellow, black distally. Endites yellow with black stripe proximally. Sternum yellow with somewhat darker edges. Dorsal part of opisthosoma yellow with numerous light brown and dark brown irregular spots. Lateral sides of opisthosoma yellow with numerous small brown spots. Ventral part of opisthosoma light yellow with some tiny light brown spots. Each side of opisthosoma with c. 19 triangular tubercles, few of them with a seta on the top. Spinnerets light brown.

Eye sizes and interdistances: AME 0.32, ALE 0.28, PME 0.30, PLE 0.24; AME–AME 0.96, AME–ALE 0.55, PME–PME 1.46, PME–PLE 0.80, AME–PME 0.62, ALE–PLE 0.53. Clypeus height at AME 0.77, at ALE 0.86.

Leg and palp measurements: Palp: 1.83, 1.36, 1.37, 1.86 (6.42). Leg I: 5.70, 1.92, 4.14, 3.21, 2.07 (17.04). Leg II: 7.25, 3.64, 5.36, 3.78, 2.32 (22.35). Leg III: 4.26, 2.41, 3.22, 1.93, 1.54 (13.36). Leg IV: 4.22, 2.19, 2.93, 1.84, 1.50 (12.68).

Palp coloration: Fe–Ti yellow with brown and dark brown irregular spots; Ta same dorsally, black with yellow tip ventrally. Legs coloration: Fe I dark brown with irregular yellow patches dorsally and retrolaterally, prolateral surface yellow with dark brown spots, yellow with dark brown spots ventrally; Fe II same as Fe I; Fe III–IV light brown with black and yellow spots on distal part. Pa I–II dark brown with irregular yellow patches; Pa III–IV dark brown with irregular yellow patches. Ti I–II dark brown with irregular yellow patches; Ti III–IV dark brown with irregular yellow patches. Mt I–II dark brown with irregular yellow patches; Mt III–IV dark brown with irregular yellow patches. Ta I–II yellow with thin black stripe dorsally; Ta III–IV yellow. Femora I and II with number of tubercles especially large at tibiae; tibiae I and II with large ventral spines (tibia I – 7; tibia II – 11).

**Figures 5–7.**
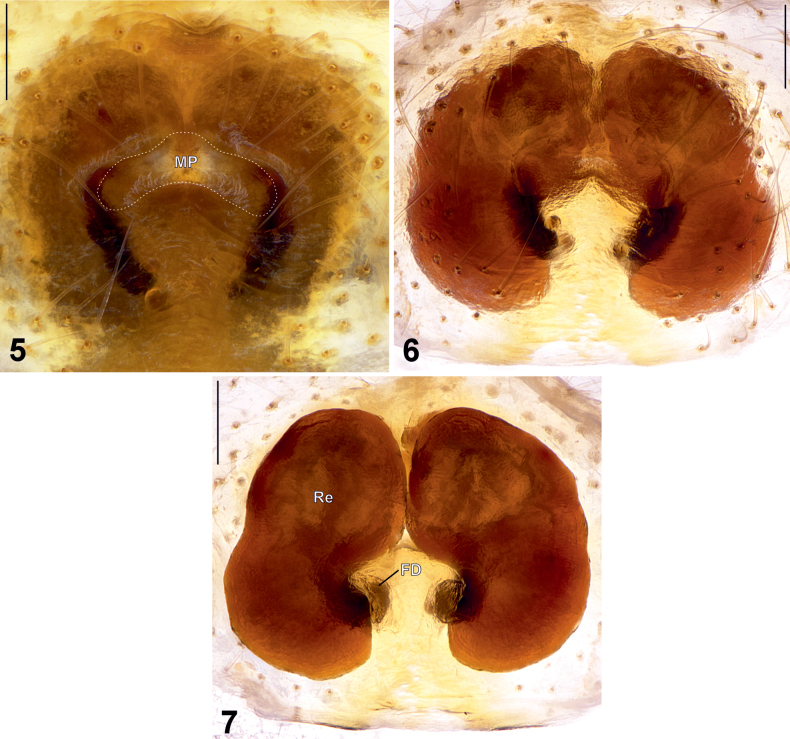
Epigyne of *Phrynarachne gorochovi* sp. nov. 5. Intact, ventral; 6. Macerated, ventral; 7. Macerated, dorsal. Abbreviations: FD = fertilization duct, MP = median plate, Re = receptacle. Scale bars: 0.2 mm.

Epigyne as shown in Figs 5–7, with inconspicuous margins, width/length ratio 1. Median plate (MP) inverted V-shaped, posterior margin indistinct and lateral edges directed posteriorly (outlined with a dashed line in Fig. 5); hood absent, but with shallow cavity; width/length ratio 2.3. Copulatory openings (CO) indistinct. Receptacles (Re) kidney-shaped, touching each other anteriorly, with smooth surface; anterior/posterior edge width ratio c. 2.5. Fertilization ducts (FD) transverse.

##### Notes.

This species represents the first published record of the genus in the Philippines. However, there are four *Phrynarachne* observations on iNaturalist (2025): one clearly belongs to *P. ceylonica*, and the others likely represent undescribed species. The only Asian *Phrynarachne* species known exclusively from a male is *P. bimaculata*, whose type locality is in Myanmar – nearly 3000 km away from the collection sites of *Phrynarachne gorochovi* sp. nov. This large geographic gap further reduces the likelihood of conspecificity.

##### Distribution.

Type locality only, Philippines (Fig. 28).

#### ﻿ Phrynarachne storozhenkoi sp. nov.

Taxon classificationAnimaliaAraneaeThomisidae

28EE7ECA-54B3-5D1A-9173-22671A15EF96

https://zoobank.org/6909DB71-5163-45EF-A000-F9FDC1BAD543

Figs 8–13, 28

##### Type material.

***Holotype*.** Malaysia • ♀; Borneo, Sabah State, environs of Tawau; 4°24'N, 117°53'E; 300 m a.s.l.; 30.08.–7.09.1994; A.M. Emelyanov leg.; ZMMU.

##### Etymology.

The specific epithet honors Sergei Storozhenko (Vladivostok, Russia), the distinguished Russian entomologist known for his studies of pygmy grasshoppers (Tetrigidae) and other orthopterans, including fossil Grylloblattodea. This name is bestowed in the year of the 70^th^ anniversary of his birth.

##### Diagnosis.

**Figures 8–13.**
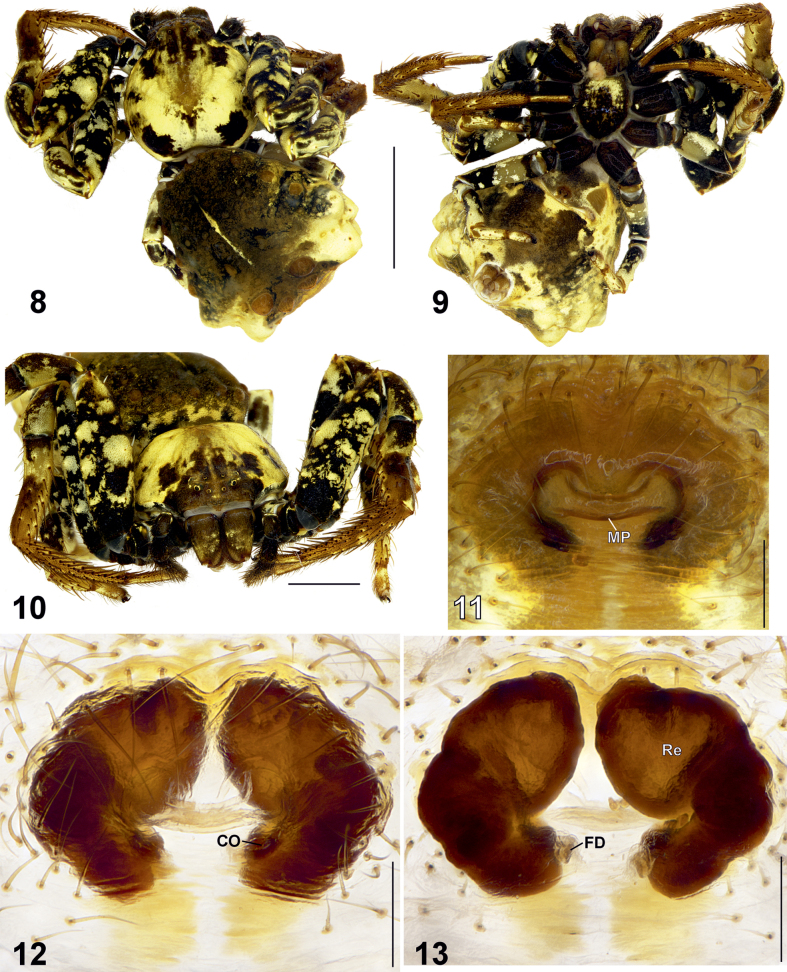
Female habitus (8–10) and epigyne (11–13) of *Phrynarachne storozhenkoi* sp. nov. 8. Dorsal; 9. Ventral; 10. Anterior; 11. Intact, ventral; 12. Macerated, ventral; 13. Macerated, dorsal. Abbreviations: CO = copulatory opening, FD = fertilization duct, MP = median plate, Re = receptacle. Scale bars: 5 mm (8, 9); 2.5 mm (10); 0.2 mm (11–13).

By the pale coloration of the carapace and opisthosoma with small, rounded tubercles, the female of *P. storozhenkoi* sp. nov. resembles those of the Asian *P. decipiens* (Forbes, 1884), *P. lancea* Tang & Li, 2010 and *P. mammillata*, known from southern China. The female of the new species can be distinguished from both similar species by the dark, almost black coloration of the dorsal side of opisthosoma (vs. white; cf. Figs 8, 20 and Lin et al. 2022: fig. 19A). From *P. decipiens*, the new species also differs by the rounded, widened lateral edges of the median plate (MP) (vs. edges pointed, narrow, directed anteriorly; cf. Figs 11, 23). From *P. lancea*, it can be distinguished by the broad and slightly curved median plate (MP) (vs. narrow and strongly curved; cf. Fig. 11, Lin et al. 2022: fig. 11A) and the receptacles (Re) nearly touching each other anteriorly (vs. widely spaced; cf. Fig. 13, Lin et al. 2022: fig. 11B). By the structure of the copulatory apparatus, the new species also resembles *P. mammillata* but can be easily distinguished from it by the smooth receptacles (vs. those covered with numerous deep folds; cf. Fig. 13, Lin et al. 2022: fig. 13B).

##### Description.

**Female** (Figs 8–10). Total length 12.61. Prosoma 5.51 long, 5.36 wide. Opisthosoma 7.62 long, 7.98 wide. Carapace yellowish white, with black head area and four irregular black spots (two anterior, two posterior). Clypeus dark brown. Chelicerae dark brown with tiny yellow spots. Labium yellowish brown, somewhat darker posteriorly. Endites yellowish brown, somewhat darker posteriorly. Sternum black with yellow n-mark anteriorly. Dorsal part of opisthosoma dark brown, with anterior half darker than posterior, bearing a thin yellow longitudinal mark and two large yellowish-white posterolateral spots. Lateral sides of opisthosoma yellowish white with irregular dark brown spots. Ventral part of opisthosoma dark brown with yellow spots. Each side of opisthosoma with 12 triangular tubercles, some with a clavate seta on the top. Spinnerets brown.

Eye sizes and interdistances: AME 0.13, ALE 0.20, PME 0.16, PLE 0.16; AME–AME 0.44, AME–ALE 0.28, PME–PME 0.49, PME–PLE 0.46, AME–PME 0.37, ALE–PLE 0.40. Clypeus height at AME 0.45, at ALE 0.56.

Leg and palp measurements: Palp: 1.38, 0.85, 0.99, 1.68 (4.90). Leg I: 5.97, 2.64, 4.12, 4.32, 1.71 (18.76). Leg II: 5.89, 2.47, 3.97, 4.09, 1.73 (18.15). Leg III: 3.29, 1.78, 2.37, 1.53, 1.14 (10.11). Leg IV: 3.53, 1.62, 2.39, 1.40, 1.00 (9.94).

Palp coloration: Fe black with thin yellow longitudinal stripe dorsally; Pa–Ti black; Ta yellowish brown with black proximal part. Legs coloration: Fe I–II black with yellow irregular spots; Fe III proximal half brown with yellow spots, distal half black with yellow spots; Fe IV brown with yellow spots. Pa I–IV black with yellow irregular spots. Ti I–II dark brown with yellow irregular spots and black spots proximally; Ti III dark brown with distal half darker than proximal and irregular yellow spots; Ti IV black with irregular yellow spots. Mt I–II light brown with tiny yellow spots; Mt III–IV yellow with light brown lateral sides. Ta I–II light brown with yellow dorsoprolateral sides; Ta III–IV light brown with yellow spots. Femora I and II with tubercles; tibiae I and II with large ventral spines (tibia I – 6; tibia II – 6).

Epigyne as shown in Figs 11–13, with M-shaped sclerotized margins, width/length ratio 1.5. Median plate (MP) broad and transversely elongate, dumbbell-shaped with smoothly concave anterior and posterior edges and rounded, widened lateral edges, forming arcuate, smile-shaped structure; hood absent; width/length ratio 3. Copulatory openings (CO) distinct. Receptacles (Re) kidney-shaped, close to each other anteriorly, with uneven surface; anterior/posterior edge width ratio c. 1.8. Fertilization ducts (FD) transverse.

##### Notes.

Although no species of *Phrynarachne* from Borneo are listed in the WSC (2025), more than 80 observations of Bornean spiders from this genus have been published on iNaturalist (2025). In addition, photographs showing the external appearance of four species (*P. ceylonica*, *P. decipiens*, *P. tuberosa* (Blackwall, 1864), and one undescribed species) were published by Koh and Bay (2019). The probability that the female of *P. storozhenkoi* sp. nov. is conspecific with *P. bimaculata*, known only from the male, is extremely low because of the large geographic gap (about 3000 km) separating the type localities of these species.

##### Distribution.

Type locality only, Borneo (Fig. 28).

#### ﻿ Phrynarachne ceylonica

Taxon classificationAnimaliaAraneaeThomisidae

(O. Pickard-Cambridge, 1884)

B9D5F0B2-4A75-5D9B-9BD4-3CA25BFD0E4B

Figs 14–19, 26, 28

Phrynarachne ceylonica : Zhu and Song 2006: 549, f. 1–5 (♂♀).
P. ceylonica : Ono 2009: 504, f. 50–55 (♂♀).
P. ceylonica : Dash and Sivaperuman 2021: 50, f. 2a–d, 3a–b (♀).^1^

##### Material examined.

Laos • 1♀; Vientiane Province, environs of Nam-Lik Eco-Village; 18°36'N, 102°24'E; 7.07.2017; M.M. Omelko leg.; hand-picking from vegetation; IBSS.

##### Diagnosis.

By the dark coloration of the prosoma and opisthosoma, combined with the white femora and patellae of the walking legs, the female of *P. ceylonica* resembles those of *P. katoi* Chikuni, 1955 and *P. xuxiake* Lin & Li, 2022. It can be easily distinguished from the former by the presence of an epigynal hood (H) (vs. absent; cf. Fig. 19 and Ono 1988: fig. 23). From *P. xuxiake*, it differs by the width /length ratio of the median plate, which is 2.9 (vs. 2.5; cf. Fig. 17 and Lin et al. 2022: fig. 14A).

##### Description.

**Figures 14–19.**
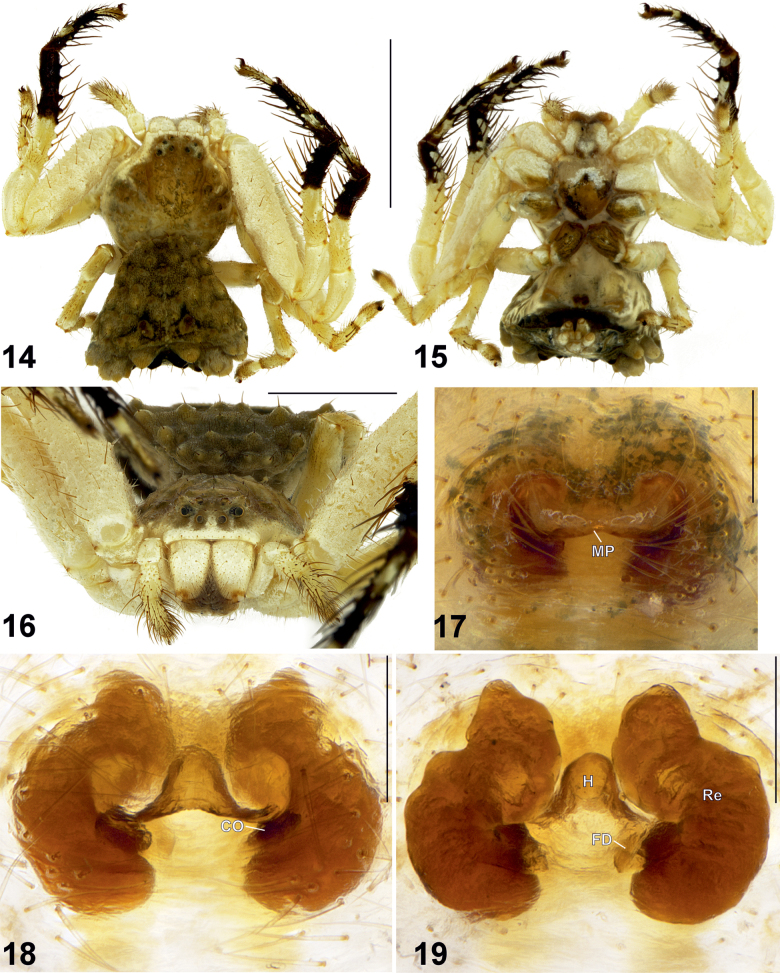
Female habitus (14–16) and epigyne (17–19) of *Phrynarachne ceylonica.* 14. Dorsal; 15. Ventral; 16. Anterior; 17. Intact, ventral; 18. Macerated, ventral; 19. Macerated, dorsal. Abbreviations: CO = copulatory opening, FD = fertilization duct, H = hood, MP = median plate, Re = receptacle. Scale bars: 5 mm (14, 15); 2.5 mm (16); 0.2 mm (17–19).

**Female** (Figs 14–16, 26). Total length 7.28. Carapace 3.85 long, 3.69 wide. Opisthosoma 4.22 long, 4.80 wide. Carapace brown without pattern, with yellow edges anteriorly. Clypeus labium yellow and endites yellow. Chelicerae yellow. Sternum light brown with yellow spot. Dorsal part of opisthosoma brown without pattern. Lateral sides of opisthosoma yellowish with small brown spots. Ventral part of opisthosoma brown anteriorly, black posteriorly. Each side of opisthosoma with c. 15 triangular tubercles, each with a long seta on the top. Spinnerets light brown.

Eye sizes and interdistances: AME 0.15, ALE 0.18, PME 0.11, PLE 0.17; AME–AME 0.32, AME–ALE 0.18, PME–PME 0.39, PME–PLE 0.36, AME–PME 0.33, ALE–PLE 0.33. Clypeus height at AME 0.31, at ALE 0.46.

Leg and palp measurements: Palp: 1.07, 0.49, 0.67, 1.09 (3.32). Leg I: 4.87, 2.10, 4.00, 2.63, 1.46 (15.06). Leg II: 5.04, 1.98, 3.69, 2.48, 1.45 (14.64). Leg III: 2.71, 1.20, 1.64, 0.96, 0.95 (7.46). Leg IV: 2.79, 1.16, 1.81, 1.12, 0.93 (7.81).

Palp coloration: Fe and Pa yellowish white; Ti light brown; Ta light brown. Legs coloration: Fe I–II yellowish white with somewhat darker dorsal side, III–IV light brown. Pa I–II yellowish white; III–IV light brown. Ti I–II black half distally, yellowish-white proximally; III–IV light brown. Mt I–II black with tiny, irregular yellow spots; III–IV light brown. Ta I–IV yellow with light brown proximal part. Femora I and II with small tubercles; tibiae I and II with very long, slightly curved ventral spines (tibia I – 16; tibia II – 16).

Epigyne as shown in Figs 17–19, plate with M-shaped sclerotized margins, width/length ratio 1.3. Median plate (MP) broad and transversely elongate, with smoothly concave anterior edge and slightly arched posterior edge, lateral edges widened and directed anteriorly; hood (H) bell-shaped, clearly visible; width/length ratio 2.9 Copulatory openings (CO) distinct. Receptacles (Re) kidney-shaped, anterior portions narrow and forward-directed, widely separated from each other anteriorly, with uneven surface; anterior/posterior edge width ratio c. 1.25. Fertilization ducts (FD) diagonal.

##### Notes.

This species has the widest distribution range in Asia, extending for about 5000 kilometers from Sri Lanka in the west to Ishigaki Island (Japan) in the east.

##### Distribution.

India (Assam, Andaman and Nicobar Islands), Sri Lanka, China (Guangxi, Yunnan), Taiwan, Japan (Iriomote-jima and Ishigaki islands), Laos (new record) (Fig. 28).

#### ﻿ Phrynarachne decipiens

Taxon classificationAnimaliaAraneaeThomisidae

(Forbes, 1884)

D1E7D3A5-CA56-5DBC-B3F5-0451C37CF100

Figs 20–25, 27, 28

Thomisus decipiens : Jacobson 1921: 186, pl. 12, f. 1–4 (♀).
Phrynarachne decipiens : Das et al. 2024: 63, f. 1–10 (♀).^2^

##### Material examined.

Laos • 1 ♀; Vientiane Province, environs of Nam-Lik Eco-Village; 18°36'N, 102°24'E; 7.07.2017; M.M. Omelko leg.; hand-picking from vegetation; IBSS.

##### Diagnosis.

By the coloration of the carapace and opisthosoma, with small, rounded tubercles, the female of *P. decipiens* resembles those of *P. storozhenkoi* sp. nov. For differences between these species, see the diagnosis of the latter.

##### Description.

**Figures 20–25.**
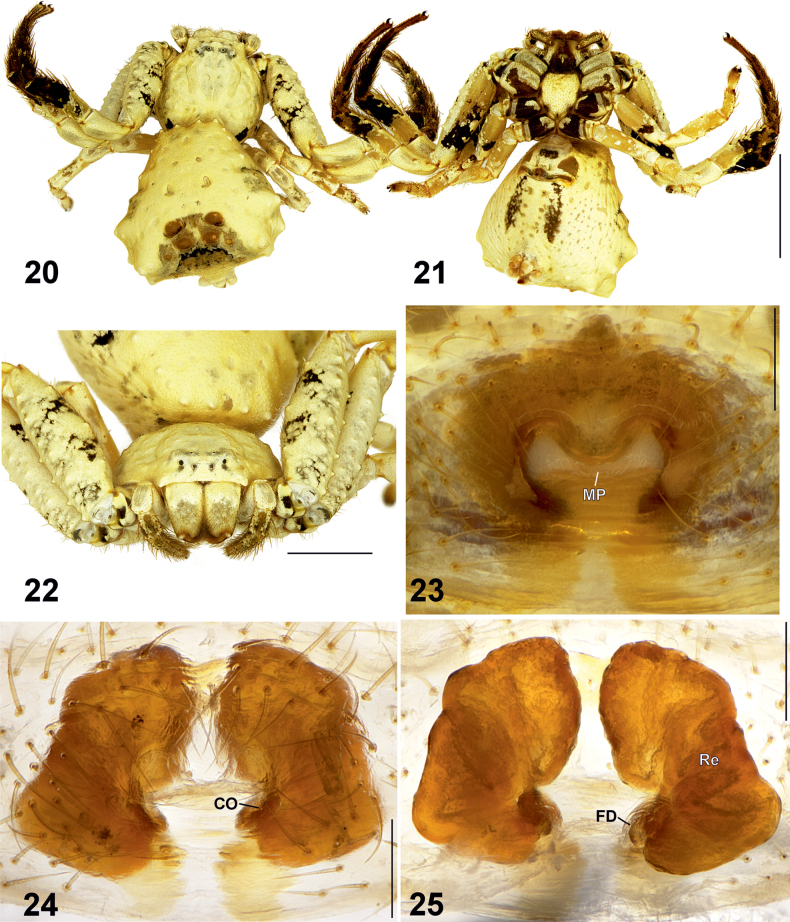
Female habitus (20–22) and epigyne (23–25) of *Phrynarachne decipiens.* 20. Dorsal; 21. Ventral; 22. Anterior; 23. Intact, ventral; 24. Macerated, ventral; 25. Macerated, dorsal. Abbreviations: CO = copulatory opening, FD = fertilization duct, MP = median plate, Re = receptacle. Scale bars: 5 mm (20, 21); 2.5 mm (22); 0.2 mm (23–25).

**Female** (Figs 20–22, 27). Total length 11.67. Carapace 4.55 long, 5.05 wide. Opisthosoma 7.62 long, 8.04 wide. Carapace yellowish white with a couple of black spots posteriorly. Clypeus yellowish white. Chelicerae light brown with yellowish-white proximal part. Labium black with yellow distal edge. Endites yellowish. Sternum yellowish white. Dorsal part of opisthosoma yellowish white with light brown spot posteriorly. Lateral sides of opisthosoma yellowish white with some brown spots. Ventral part of opisthosoma yellowish white with two longitudinal brown stripes. Each side of opisthosoma with c. 15 triangular tubercles, each with a clavate seta on the top. Spinnerets light brown.

Eye sizes and interdistances: AME 0.18, ALE 0.20, PME 0.14, PLE 0.15; AME–AME 0.35, AME–ALE 0.21, PME–PME 0.53, PME–PLE 0.46, AME–PME 0.35, ALE–PLE 0.33. Clypeus height at AME 0.36, at ALE 0.47.

Leg and palp measurements: Palp: 1.39, 0.95, 0.99, 1.63 (4.96). Leg I: 5.36, 2.48, 3.37, 3.88, 1.58 (16.67). Leg II: 5.48, 2.46, 3.45, 3.67, 1.69 (16.75). Leg III: 2.94, 1.73, 1.81, 1.35, 1.17 (9.00). Leg IV: 3.10, 1.50, 2.28, 1.39, 1.02 (9.29).

Palp coloration: Fe and Pa yellowish white; Ti yellowish white with brown spots; Ta brown. Legs coloration: Fe I–II yellowish white with black irregular spots, III–IV yellowish white with brown spots. Pa I–IV yellowish white with darker dorsal side. Ti I–II black half distally, yellowish-white proximally; III–IV yellowish white with irregular brown spots. Mt I–II dark brown with tiny yellow spots; III–IV yellowish white with irregular brown spots. Ta I–II brown with yellow prolateral side; III yellow with brown distal part; IV yellow. Femora I and II with small tubercles; tibiae I and II with large ventral spines (tibia I – 8; tibia II – 7).

**Figures 26–27.**
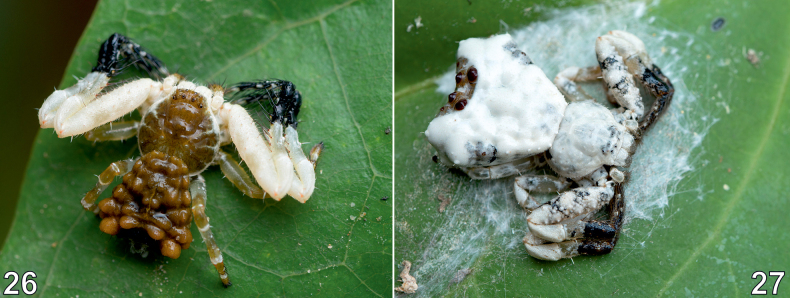
Living female of *Phrynarachne ceylonica* (26) and *P. decipiens* (27) in situ.

**Figure 28.**
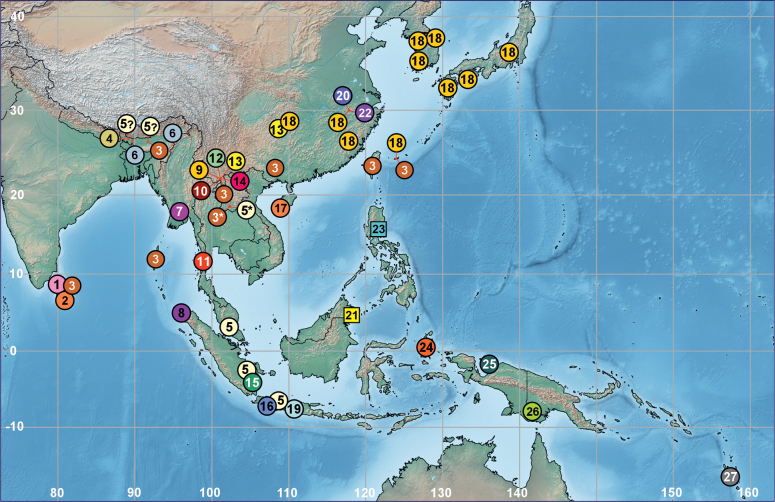
Distribution records of *Phrynarachne* spp. in Asia and Australasia: *P. fatalis* (1), *P. rothschildi* (2), *P. ceylonica* (3), *P. tuberosa* (4), *P. decipiens* (5), *P. peeliana* (6), *P. bimaculata* (7), *P. papulata* (8), *P. brevis* (9), *P. lancea* (10), *P. aspera* (11), *P. dreepy* (12), *P. mammillata* (13), *P. zhengzhongi* (14), *P. kannegieteri* (15), *P. dissimilis* (16), *P. yunhui* (17), *P. katoi* (18), *P. coerulescens* (19), *P. huangshanensis* (20), *P. storozhenkoi* sp. nov. (21), *P. xuxiake* (22), *P. gorochovi* sp. nov. (23), *P. cucullata* (24), *P. jobiensis* (25), *P. tuberculata* (26), *P. cheesmanae* (27). New species are marked with squares; new records with asterisks; dubious records with question marks.

Epigyne as shown in Figs 23–25, with M-shaped sclerotized margins, width/length ratio 1.3. Median plate (MP) broad and transversely elongate, with smoothly concave anterior and posterior edges, lateral edges widened and directed anteriorly; hood clearly absent; width/length ratio 2.7. Copulatory openings (CO) distinct. Receptacles (Re) kidney-shaped, closely placed to each other anteriorly, with uneven surface, anterior parts almost parallel mesally; anterior/posterior edge width ratio c. 1.75. Fertilization ducts (FD) diagonal.

##### Notes.

See the information on the distribution of this species in the Discussion section.

##### Distribution.

India, Malaysia (Malay Peninsula), Indonesia (Java, Sumatra) and Laos (new record) (Fig. 28).

## ﻿Discussion

More than half of all known *Phrynarachne* species are described based solely on females (WSC 2025). Fortunately, the copulatory organs of many of these are well illustrated, which allows confident differentiation of the two new species described herein from the majority of known taxa. However, six Asian species: *P. coerulescens* (Doleschall, 1859), *P. cucullata* Simon, 1886, *P. dissimilis* (Doleschall, 1859), *P. kannegieteri* van Hasselt, 1893, *P. papulata* Thorell, 1891, and *P. rothschildi* Pocock, 1903 are known only from females and lack any published illustrations of the epigyne. Among them, *P. coerulescens*, *P. dissimilis*, and *P. rothschildi* are accompanied by illustrations of general habitus, which clearly differ from those of the new species and rule out conspecificity. The remaining three species are known only from textual descriptions. The original descriptions of *P. kannegieteri* and *P. papulata* provide relatively detailed accounts of female morphology, which differ markedly from the females of *P. gorochovi* sp. nov. and *P. storozhenkoi* sp. nov., thereby excluding conspecificity.

*Phrynarachne decipiens* was first collected on the banks of the Moesi River in southern Sumatra (Forbes 1884). In the same paper, the author also mentions, without specifying an exact locality, that he had previously encountered the species in western Java. Forbes provided a description of the spider’s web, which mimics bird droppings, and included a color illustration showing a specimen resting in the web on a leaf. Notably, Forbes (1884) also claimed—likely in error—that the spider attaches itself to the web in an inverted position, with the dorsal side attached to the silk. Shortly afterward, O. Pickard-Cambridge (1884) published a high-quality black-and-white drawing of a specimen collected by Forbes, although no illustration of the female copulatory organ was provided. The drawing clearly depicts the distinctive coloration of legs I and II, the carapace, and the dorsal surface of the opisthosoma. Later, the epigyne of a female collected by Forbes in Java was illustrated by Workman (1896). Although schematic, the drawing shows a well-defined median plate with a curved anterior margin.

Subsequently, *P. decipiens* was recorded from mainland Malaysia (Mount Ophir) by Jacobson (1921), who published two illustrations of spiders under this name. One of them (Jacobson 1921: fig. 1) corresponds well to the descriptions provided by the earlier authors and matches my specimen from Laos, while the other (Jacobson 1921: fig. 4) clearly depicts a female of another species of this genus. More recently, this species was reported from the Indian state of Assam (Das et al. 2024). In that paper, the authors provided photographs of both the external morphology and the copulatory organs. It is noteworthy that the first two pairs of legs in the Indian specimens are much lighter than those illustrated in earlier studies and in my specimen. Moreover, the shape of the receptacles in Indian females differs markedly from that observed in my specimen (cf. Figs 24–25 and Das et al. 2024: figs 6–9). In summary, although the type specimens of *P. decipiens* have not been examined by modern authors and are possibly lost, the characteristic body coloration strongly suggests that my specimen is conspecific with those collected in Southeast Asia by Forbes (1884) and Jacobson (1921). In contrast, the records from northern India (Das et al. 2024) may represent a different, possibly undescribed species of *Phrynarachne*.

## Supplementary Material

XML Treatment for Phrynarachne

XML Treatment for Phrynarachne gorochovi

XML Treatment for Phrynarachne storozhenkoi

XML Treatment for Phrynarachne ceylonica

XML Treatment for Phrynarachne decipiens

## Footnotes

For the full list of references (ten entries) see WSC (2025).

For the full list of references (six entries), see WSC (2025).

## ﻿Additional information

### Use of AI

No use of AI was reported.

### Author ORCIDs

Mikhail M. Omelko https://orcid.org/0000-0002-1556-6248

### Data availability

All of the data that support the findings of this study are available in the main text.
